# Toxicity of Landfill Leachate to Stream-Dwelling Benthic Macroinvertebrates

**DOI:** 10.3390/toxics14020109

**Published:** 2026-01-23

**Authors:** Neal D. Mundahl, Erik D. Mundahl

**Affiliations:** 1 Program in Ecology and Environmental Science and Large River Studies Center, Department of Biology, Winona State University, Winona, MN 55987, USA; 2 Mundahl Nordic Services, Eagle River, AK 99577, USA

**Keywords:** landfill leachate, toxicity, benthic macroinvertebrates, *Brachycentrus*, trout streams

## Abstract

Laboratory and field investigations were used to assess the toxicity of leachate from a closed sanitary landfill on benthic macroinvertebrates in coldwater trout streams located near a landfill in southeastern Minnesota, USA. Field-collected invertebrates were exposed to a range of concentrations (0–100%) of leachate during a series of 24 h and 7-day laboratory toxicity tests. Benthic macroinvertebrates also were collected from two stream sites on either side of the landfill and at a third site downstream to assess potential pollution exposure of the stream communities. Ten different taxa exposed to 100% leachate for 24 h exhibited survival ranging from 0 to 100%, with survivorship not correlated to published invertebrate pollution tolerance values. More extensive 24 h tests with the least tolerant *Brachycentrus* caddisfly larvae found 100% mortality at leachate concentrations > 70%, with the first mortalities observed after 3 h. *Brachycentrus* had 100% survival at leachate concentrations < 40%. During 7-day tests, *Brachycentrus* had 100% survival at all leachate concentrations of 40% and lower, but all *Brachycentrus* died after 2 days at concentrations of 60% and higher. Instream benthic communities, averaging 12 to 17 different taxa at the various stream sites, were rated from good to excellent based on biotic index values, with intolerant taxa present at all three stream sites. Landfill leachate has not impacted the benthic invertebrate communities in streams nearby, but leachate at higher concentrations has the potential to be toxic to a variety of local taxa.

## 1. Introduction

Approximately 50% of municipal solid wastes (MSW) produced in the USA end up in sanitary landfills [1]. Landfills have transitioned over time from simple dumping grounds for the disposal of unwanted refuse to highly engineered systems designed to facilitate the decomposition of organic waste while protecting the environment from potentially harmful chemicals and materials. This transition was driven largely by a series of environmental regulations established at the national level that restricted and regulated the handling and disposal of most types of solid wastes [1]. Although finding locations for new landfills to replace filled and closed sites is becoming increasingly difficult, landfills likely will continue to serve an important role in MSW management well into the future.

One side effect of landfill operations is the production of leachate, a complex solution of chemicals resulting from the decomposition of organic matter and rainfall percolating through layers of mixed MSW within a landfill [2,3,4,5]. Leachate is produced in both active and closed landfills; the volume produced decreases as decomposition slows and filled landfills are capped to reduce or prevent further infiltration of rainwater [2]. The chemical composition of leachate can be highly variable depending on the types of materials landfilled and the quality (e.g., acidity, wash-out of airborne pollutants) of the rainwater itself. Generally, leachate contains large amounts of dissolved organic matter, salts, heavy metal ions, and a variety of other organic compounds [2,3,4,5]. To manage leachate, contemporary landfills are required: (1) to be underlain by synthetic liners to prevent leachate from seeping into underground aquifers, and (2) to have an operating leachate collection system to siphon off accumulating leachate from the liners and direct it to wastewater treatment facilities [6,7].

Landfill leachate can be highly toxic to a variety of aquatic life forms, ranging from bacteria and algae to plants, invertebrates, and fish [4,8,9,10,11,12,13,14]. Previous studies have suggested that this toxicity results from the various salts, heavy metals, and volatile organic chemicals present within typical leachate, either separately or in combination [4,11,14]. High levels of un-ionized ammonia and alkalinity (i.e., high pH) have been identified frequently as the most toxic components of landfill leachate [4,8,9,10,11,12,13]. In some instances, various treatment approaches may be used to break down and detoxify landfill leachate [2,4,5,15]. This toxicity is why regulatory agencies, such as the United States Environmental Protection Agency, require regular monitoring of groundwater and surface waters nearby both active and closed landfills.

Previous bioassays of landfill leachate have used bacteria, aquatic plants, plant seeds and cells, several types of zooplankton, and fish [8,9,10,11,12,13,14,16,17,18,19,20]. Variable results have been reported even when using the same test species, sometimes attributed to variation in the composition of the leachate being tested [4,8,10]. Some studies also have reported differing toxicities in leachate collected from active versus closed landfills [3,13]. Consequently, although all studies have reported that landfill leachate is toxic to various aquatic organisms, there has been no consensus on what concentration of leachate typically is lethal to 50% of the organisms tested (LC50) after 24, 48, or 96 h [4,8,9,10,11,12,14]. There also has been little to no bioassay testing of naturally occurring and abundant benthic invertebrates that could be impacted if leachate leaked from a local landfill into nearby waterways [4].

In this study, we took two approaches to examine the potential toxicity of landfill leachate to aquatic organisms. First, we conducted laboratory bioassays of leachate from a local closed landfill, using a variety of locally collected stream-dwelling organisms encompassing a range of assumed pollution tolerance levels, followed by additional tests with the most sensitive type. Second, we assessed and compared the natural communities of benthic macroinvertebrates among three stream sites near the landfill to examine potential exposures and vulnerabilities of these communities to water-borne pollutants (e.g., leachate or other potential physical/chemical stressors). We hypothesized that the toxicity of leachate to various invertebrate taxa would correspond to their reported tolerance levels (i.e., less tolerant species would suffer higher mortality when exposed to leachate, whereas more tolerant species would experience lower mortality), and that the most sensitive species would display increasing mortality rates with increasing leachate concentrations. We also hypothesized that benthic macroinvertebrate assemblages at stream sites nearest the landfill would exhibit higher community tolerance levels (i.e., more tolerant of pollution) and reduced community diversity and integrity than those further downstream from the landfill.

## 2. Materials and Methods

### 2.1. Winona County Landfill and Nearby Streams

**Figure 1:**
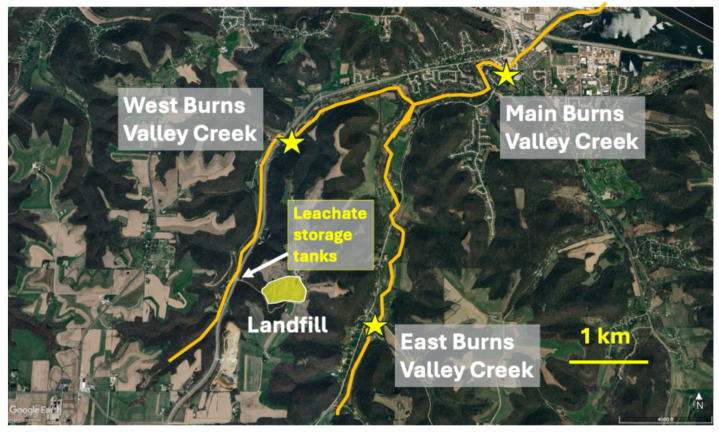
Aerial view of the Burns Valley Creek study area in southeastern MN, USA, indicating locations of the landfill, leachate storage tanks, and benthic macroinvertebrate collection sites (yellow stars) in WBVC, EBVC, and MBVC. Background photo from GoogleEarth.

The Winona County landfill in rural southeastern Minnesota, USA (43°59′53.27″ N, 91°39′45.99″ W) began operations in the early 1970s as a private disposal facility accepting municipal solid waste, demolition waste, and industrial/hazardous waste (with permission from the State of Minnesota) from nearby industries. It is located on former agricultural land on a high ridge (376 m above MSL) separating two stream valleys (East Burns Valley, West Burns Valley), 114 m and 144 m below the ridge (Figure 1). The original facility consisted of a clay-lined pit lacking methane venting and leachate collection systems. After purchase by Winona County in 1983, industrial/hazardous wastes were removed, and multiple lined cells were constructed. The landfill closed in 1998 after the lined cells were filled and long-term care of the site was transferred to the State of Minnesota (Minnesota Pollution Control Agency) in 2000. Between 2005 and 2007, the State moved most of the waste from old, unlined disposal cells to a new, lined cell equipped with methane venting and leachate collection systems, leaving only 3 hectares of unlined wastes.

Leachate collected from the landfill originally was piped via a gravity-flow, underground pipeline through West Burns Valley to the regional wastewater treatment facility. After the pipeline became clogged beyond repair, leachate storage tanks were installed on-site, with leachate then regularly trucked to the treatment facility. Leachate used in this study was obtained from these storage tanks during September (late summer/early autumn; Figure 1).

West Burns Valley Creek (WBVC) and East Burns Valley Creek (EBVC) are both 1st to 2nd-order coldwater streams that flow northward for 6 and 7 km, respectively, in valleys flanking the ridge upon which the Winona County landfill is located (Figure 1). Both streams arise from multiple groundwater springs in their upper reaches. These streams converge 2.7 km northeast of the landfill to form the 3rd-order Main Burns Valley Creek (MBVC), which flows 3.3 km through the City of Winona into the backwaters of the Mississippi River. The entire stream system is a state-designated trout habitat, containing native brook trout (*Salvelinus fontinalis*) in the upper sections of both East and West Burns, and naturalized brown trout (*Salmo trutta*) throughout the system. The stream system also contains slimy sculpin (*Uranidea cognata*), western blacknose dace (*Rhinchthys obtusus*), American brook lamprey (*Lethenteron appendix*), mud darter (*Etheostoma asprigene*), and northern pike (*Esox lucius*).

### 2.2. Benthic Macroinvertebrate Community Assessments

The communities of benthic macroinvertebrates inhabiting EBVC, WBVC, and MBVC were assessed during September/October by collecting triplicate samples from riffles in each stream (Figure 1). To collect each sample, a D-frame aquatic dip net (500-micron mesh) was positioned on the stream bottom and an area of coarse substrates approximately 0.1 m^2^ in area upstream of the net was disturbed for approximately 30 s. The current then carried dislodged organisms downstream into the net. For each sample, this procedure was followed twice, once in a slow area of the riffle and once in a fast area of the same riffle, with organisms collected from both slow and fast areas combined to form a single composite sample. No attempts were made to sample organisms from other instream habitats (e.g., fine bottom sediments, submerged logs, leafpacks and other accumulations of detritus, overhanging submerged streambank vegetation). Samples were preserved in 70% ethanol prior to sorting and identification (primarily to genus) of all organisms in the lab using local and regional identification keys [21,22,23,24,25].

Once identified, each macroinvertebrate taxon was assigned a pollution tolerance value (ranging from 0 to 10, where 0 = very intolerant and 10 = very tolerant) based on previous studies [21,22,23,24,25,26,27]. Hilsenhoff’s biotic index [26,27] was then calculated for each benthic macroinvertebrate sample using each taxon’s tolerance value and its relative abundance in the sample. Biotic index values for each sample were assessed based on the following criteria: 0–3.5 = no apparent organic pollution; 3.51–4.5 = possible slight organic pollution; 4.51–5.5 = some organic pollution; 5.51–6.5 = fairly significant organic pollution; 6.51–7.5 = significant organic pollution; 7.51–8.5 = very significant organic pollution; and 8.51–10.0 = severe organic pollution) [27]. Biotic index values were compared among the three streams to assess potential degrees of exposure of the benthic communities to organic pollution.

In addition, a regional, multi-metric benthic invertebrate biotic integrity index (B-IBI) [28,29] was calculated for each of the macroinvertebrate samples from the three Burns Valley Creek stream sites as an indicator of benthic biological integrity for the sites. Index scoring (possible scores from 0 to 100) was based on values for 10 different metrics that included various forms of taxa richness, tolerance to environmental stressors, feeding strategies, and longevity measures, and produced ratings such as: excellent (65–100), good (50–60), fair (30–45), poor (10–25), and very poor (0–5) [22].

### 2.3. Laboratory Testing

For toxicity testing trials, various aquatic invertebrate taxa were selected from among those collected from coarse substrates in Burns Valley Creek, as well as those collected from submerged streambank vegetation. Organisms were either hand-picked from rocks lifted from the stream bottom or they were removed from the D-frame net after sweeping the net through submerged vegetation and accumulations of detritus. Captured invertebrates were placed into a cooler filled with stream water for transport to the lab. In the lab, organisms were counted and sorted according to taxon and placed into containers filled with aerated stream water to acclimate to lab conditions (see below). Test organisms with a wide range of tolerance levels (see above) were chosen, and leachate was obtained from the landfill and refrigerated in a sealed, non-aerated container until use. In addition, stream water was collected and aerated prior to use in toxicity testing.

A series of five lab tests were conducted to assess the effects of landfill leachate on local stream macroinvertebrates. All tests were conducted at 15–16 °C, 12 h light: 12 h dark light cycles, in acid-washed 1 L test containers containing aerated leachate and/or stream water. Water temperatures during testing were within 2 °C of stream temperatures during collections, and the lab photoperiod was the same as natural outdoor conditions. A small, sterilized rock was added to each test container to serve as a stable perch for macroinvertebrates. Macroinvertebrates and stream water were collected just prior to each test, with organisms allowed to acclimate to lab conditions for 24 h prior to testing. The five lab tests included:

- Twenty-four h survival tests using 10 different taxa (insects and non-insect arthropods) and 100% leachate. Mortality checked after 24 h.
- Twenty-four survival tests using *Brachycentrus* caddisfly larvae and various leachate concentrations (0, 10, 20, 30, … 100%). Dilutions were prepared using field-collected stream water. Ten larvae were exposed to each leachate concentration. Mortality checked after 24 h. Experiment conducted twice. *Brachycentrus* larvae were selected for this and subsequent tests due to its sensitivity to pollutants (Table 1) and its high abundance within the Burns Valley Creek system.
- Seven-day survival tests using *Brachycentrus* caddisfly larvae and various leachate concentrations (0, 10, 20, 30, … 100%). Ten larvae were exposed to each leachate concentration. Mortality checked daily for 7 days.
- Twenty-four h survival tests using *Brachycentrus* caddisfly larvae and leachate concentrations of 70 and 100%. Ten larvae were exposed in each of six replicates at each leachate concentration plus controls. Mortality checked hourly for 13 h, then after 24 h.
- Twenty-four h survival tests using *Brachycentrus* caddisfly larvae and 100% leachate (pre-aerated for 24 h prior to use to potentially reduce toxicities [2] versus not pre-aerated). Ten larvae were exposed in each of six replicates for each treatment plus controls. Mortality checked after 24 h. Mortality checked after 24 h.

### 2.4. Statistical Analyses

Various statistical tests were used to analyze the field and lab data generated during this study. First, a series of non-parametric Kruskal–Wallis tests were used to compare macroinvertebrate community variables (density, taxa richness, Hilsenhoff biotic index, B-IBI) among the three study sites. These tests were conducted with the aid of an online statistical calculator (VassarStats: Website for Statistical Computation; vassarstats.net (Accessed 14 October 2025)). LC50 values were calculated separately for 24 h, 48 h, 96 h, and 7-day leachate toxicity tests on *Brachycentrus* via probit analysis with a computational tool developed by [30].

## 3. Results

### 3.1. Benthic Macroinvertebrate Community Assessments

In total, 4335 organisms representing 28 taxa of benthic macroinvertebrates were collected from coarse substrates at the three stream sites (Table 1). Nine taxa were found at every site, with *Brachycentrus* caddisflies, isopods, *Optioservus* beetles, and *Baetis* mayflies collectively comprising over 76% of all organisms collected. Mean densities ranged from 1800 to 3600 individuals/m^2^ but did not differ significantly among stream sites (Table 2). Sample taxa richness ranged from nine to 17 taxa, with taxa richness highest in WBVC and differing significantly among sites (Table 2). The various taxa had tolerances that ranged from very intolerant (value of 0 for *Glossosoma* and *Rhyacophila* caddisflies) to moderately tolerant (value of 8 for isopods, Sphaeriidae clams, and *Physella* and *Amnicola* snails). No taxa listed as very tolerant (value of 9 or 10) were collected.

Hilsenhoff biotic index scores differed significantly among the three stream sites (Table 2). As hypothesized, MBVC had the lowest score, indicative of no apparent organic pollutant exposure. By contrast, both WBVC and EBVC had higher biotic index scores, indicative of possible slight and some organic pollution, respectively. However, scores for all sites fell within the good to excellent range, with little obvious impairment of benthic invertebrate communities due to exposure to organic pollutants.

B-IBI ratings of the three stream sites presented a community assessment differing dramatically from Hilsenhoff biotic index ratings (Table 2). B-IBI scores indicated that benthic communities were significantly impaired at all sites, with ratings ranging from fair/good to poor. All sites lacked expected numbers of stonefly (Plecoptera) taxa and expected proportions of predatory and long-lived taxa. Sites (WBVC, EBVC) nearest to the landfill had significantly higher B-IBI scores than the site (MBVC) furthest from the landfill. These differences resulted predominantly from more total taxa, more caddisfly (Trichoptera) taxa, and more filter-feeding taxa at WBVC and EBVC than at MBVC.

### 3.2. Landfill Leachate Chemical Analyses

The Winona County landfill leachate used in this study was analyzed by a certified testing laboratory immediately prior to toxicity testing. The leachate had a cloudy gray-brown appearance, and contained a mixture of salts, heavy metals, and volatile organics, with high alkalinity and specific conductance (Table 3). Ionized salt (e.g., calcium, magnesium, potassium, and sodium chlorides and sulfates), ammonium-nitrogen, and total dissolved solids concentrations were very high. Only four of the 68 total volatile organic compounds screened were present at detectable levels.

### 3.3. Leachate Toxicity Tests

Initial screening tests of the toxicity of landfill leachate to field-collected stream invertebrates revealed a wide range of toxicities among the 10 taxa tested (Table 4). Five taxa exhibited complete mortality after 24 h exposure to 100% (raw) leachate, whereas three taxa demonstrated no mortality. The remaining taxa showed intermediate levels of mortality. There was no significant relationship (simple linear regression: *F*_1,8_ = 0.019, *p* = 0.895, *r*^2^ = 0.002) between published invertebrate tolerance levels and % mortality when exposed to raw leachate.

The 24 h toxicity tests of landfill leachate on *Brachcentrus* caddisfly larvae demonstrated that all leachate concentrations ≥ 70% were lethal to all larvae (Table 5). No larvae died at leachate concentrations ≤ 40% (including no mortalities in control groups, 0% leachate), whereas some larvae died at concentrations of 50 and 60%.

**Figure 2:**
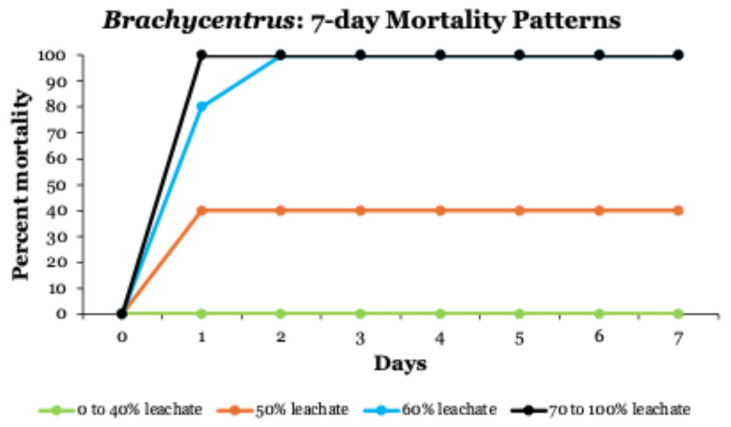
Mortality patterns of *Brachycentrus* caddisfly larvae during 7-day toxicity testing at leachate concentrations between 0 and 100%.

In 7-day toxicity tests, patterns of *Brachycentrus* mortality were like those in the 24 h tests (Figure 2). All larvae exposed to leachate concentrations ≥ 70% died within the first 24 h, whereas larvae in leachate treatments ≤ 40% survived for the entire 7-day test period (including no mortalities in control groups, 0% leachate). All larvae in 60% leachate died by 48 h, whereas only 40% of larvae in 50% leachate had died after 7 days. Based on both the 24 h and 7-day tests, LC50 concentrations for *Brachycentrus* caddisfly larvae exposed to Winona County landfill leachate varied between 46 and 55% leachate across the various time intervals examined (Table 6).

**Figure 3:**
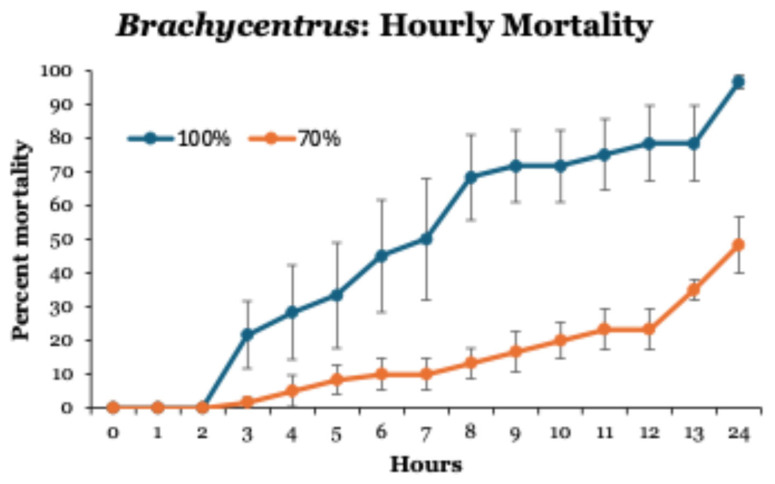
Hourly patterns of mortalities of *Brachycentrus* caddisfly larvae exposed to leachate concentrations of 70 and 100% for 24 h. Values are means ± one standard error of six tests at each concentration. Control groups not shown (no mortality in 0% leachate).

When *Brachycentrus* larvae exposed to 70 and 100% leachate were monitored hourly, no mortalities occurred during the first 2 h (Figure 3). Thereafter, mortalities reached ~70% after 8 h in 100% leachate, but only 20% after 10 h in 70% leachate. Unlike all previous tests at these concentrations, not all larvae died after 24 h of exposure.

**Figure 4:**
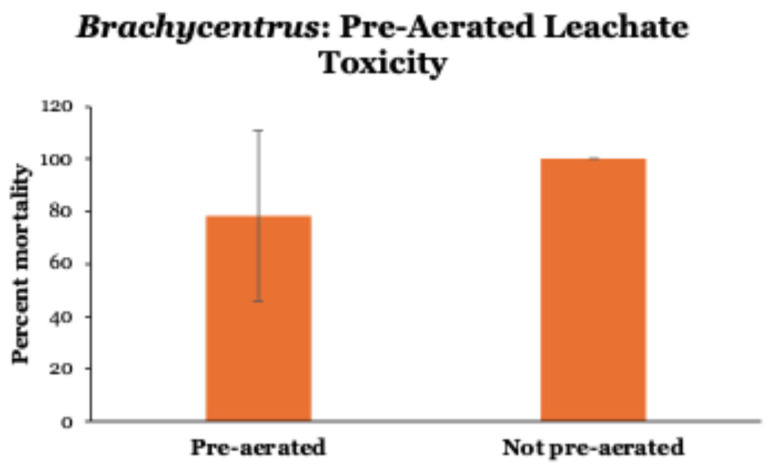
Toxicity of pre-aerated versus not pre-aerated leachate (100%) to *Brachycentrus* caddisfly larvae over 24 h. Values are means ± one standard deviation of six tests of each type. Control groups not shown (no mortality in 0% leachate, pre-aerated and not pre-aerated stream water only).

By pre-aerating 100% leachate before testing, mean 24 h mortalities of *Brachycentrus* were reduced by ~20% (Figure 4). However, due to wide variability (range from 20 to 100%) among pre-aerated tests, this reduction in mortality was not significantly different (Mann–Whitney *U* = 27, *p* = 0.174) than the 100% mortality observed in all tests conducted without pre-aerating leachate prior to use. Control groups (0% leachate, pre-aerated and not pre-aerated stream water only) displayed no mortality during the 24 h experiment.

## 4. Discussion

Leachate from the Winona County landfill, like that from so many other sanitary municipal landfills around the world, was a complicated mixture of dissolved organic matter, salts, heavy metal ions, and many other substances [2,3,4,5]. The field and laboratory studies described here resulted in four key findings about landfill leachate and its effects on aquatic invertebrates, both at organismal and community levels. First, local stream invertebrate communities appear largely unaffected by the landfill located within the watershed, even though landfilled municipal wastes previously were stored in unlined pits for many years, with some small portion of the landfill remaining in that state even to the present day. Second, aquatic invertebrates exhibited widely variable responses when exposed to raw landfill leachate, from no effect to 100% mortality, with responses unrelated to the species’ reported pollution tolerance levels. Third, diluting leachate significantly reduced its toxicity to sensitive *Brachycentrus* caddisfly larvae, with leachate EC_50_ concentrations of 45 to 55% for exposure times from 24 h up to 7 days. Finally, aerating leachate prior to its exposure to aquatic invertebrates potentially may reduce its toxicity.

The Winona County landfill is located in a region underlain by karst geology [31]. Fractured limestone and porous sandstone bedrock allow rainfall (and other substances at or near the surface) to seep into and recharge underground aquifers. These aquifers are the water sources for springs that feed the coldwater trout streams throughout the region [31]. Water quality within those springs and streams is strongly influenced by land use and subsurface geology, with stream invertebrate communities responding (by displaying more pollution tolerant taxa) to agricultural chemicals and other substances that can enter aquifers and then discharge into surface spring-fed streams [32]. The low Hilsenhoff biotic index and the presence of few tolerant taxa in Burns Valley Creek suggest that leachate from the Winona County landfill has not seeped into the groundwater aquifers nearby and found its way into the creek system. This is good news, based on the lack of leachate barriers and collection systems during the landfill’s early years, and the fact that some waste remains today on-site in cells lined only with clay. The diffuse springs (numerous, small-volume seeps emanating from aquifers with long water residence times) characteristic of the landfill area contrast with the conduit springs (small number of higher-volume springs emanating from aquifers with short water residence times) elsewhere within the region [31,32]. Diffuse springs and their connected aquifers appear to better insulate streams and their biota from watershed activities, whereas conduit springs and their aquifers can more quickly convey watershed pollution into surface streams, impacting biota directly and likely selecting for more tolerant taxa [32].

The Hilsenhoff biotic index is based solely on the documented or estimated tolerances of aquatic invertebrates to organic pollution [26,27], and our study of Burns Valley Creek found the expected pattern (if landfill leachate was influencing the stream communities) of a more tolerant invertebrate community at stream sites nearest the landfill compared to the site further downstream. More recently developed bioassessment indices are still based somewhat on the premise that taxa have differing tolerances to various stressors, assuming that low-quality or stressful sites will be dominated by tolerant species [33,34,35]. However, pollution tolerance is not the sole factor used most frequently today in aquatic bioassessment, with various indices now usually being multi-metric, using some combination of tolerance, taxa richness, feeding strategies, and longevity to quantify stream system impairment [28,35,36]. Different stressors (e.g., high or low temperatures, suspended sediments, various inorganic chemicals) may produce different changes in the aquatic community than those resulting from organic pollution [36]. Consequently, multi-metric indices like the B-IBI used here may produce different overall results than the Hilsenhoff biotic index, as evidenced in the present study, with invertebrate communities at stream sites nearest the landfill exhibiting less impairment than the site further away. By scoring and rating multiple (usually five or more) community characteristics rather than just one, multi-metric indices like the B-IBI should be more responsive to a wider variety of stressors than just organic pollution [34,35,36] and likely produce a more comprehensive picture of exposure of the stream communities to pollutants and other disturbances.

Previous studies of landfill leachate have reported toxic effects on a diversity of different test organisms [3,4,8,9,10,11,12,13,14]. However, most tests have involved the same organisms typically used in toxicity testing for many different substances, including bacteria (luminescent and otherwise), green algae, multiple types of zooplankton (especially *Daphnia magna*), fish (including fathead minnow *Pimephales promelas* and rainbow trout *Oncorhynchus mykiss*), and other taxa [4]. Although such standardized testing has provided a sound benchmark for assessing the potential environmental impacts of thousands of chemicals and compounds, direct application of lab results to different organisms and/or to field situations should be performed with caution. Although studies have been conducted using multiple species to assess leachate toxicity [10], most studies have used only a single test species [3,4,8,9,11,12,13,14], and none have used local aquatic taxa collected near a landfill.

Locally collected aquatic invertebrates have been used in toxicity testing for several decades, often by integrating both field and laboratory studies into experimental designs [37,38,39,40,41,42,43]. We used a variety of aquatic invertebrates collected from Burns Valley Creek during simple laboratory screening tests of the toxicity of Winona County landfill leachate. These invertebrates ranged widely in their reported pollution tolerance levels [21,22,23,24,25,26,27], and when exposed to leachate, displayed widely varying mortality rates not correlated with their pollution tolerance levels. In fact, both the most sensitive (*Brachycentrus* caddisfly larvae) and most tolerant (*Asellus* isopods) taxa tested displayed 100% mortality when exposed to raw leachate for 24 h, whereas three other taxa categorized as having intermediate tolerance experienced no mortality. Obviously reported tolerances of aquatic organisms are based on observed responses after exposures to a variety of environmental stressors [23,25,26], and it cannot be expected that organisms would exhibit similar mortalities when exposed to every potential stressor, especially chemically complex pollutants such as landfill leachate [3,8,9,10,12,14]. Additional testing of landfill leachate on a wider range of taxa, especially common forms from streams and rivers (e.g., mayflies, caddisflies, molluscs), would be helpful in better understanding the toxicity of this potential pollutant on the types of organisms most likely to be exposed to leachate.

When tested on the sensitive and most common invertebrate (*Brachycentrus* caddisfly larvae) from Burns Valley Creek, landfill leachate produced similar LC50 values (46–55% leachate concentrations) across the spectrum from acute to chronic time periods. Although raw (100%) leachate caused significant *Brachycentrus* mortality within hours, leachate diluted to 40% produced no mortality over a 7-day period. Although highly toxic in its unaltered state, somewhat modest dilution of leachate rendered it non-lethal to our most sensitive test organisms over the maximum time period (7 days) examined here. Although we did not examine possible non-lethal, but potentially important, effects of diluted leachate on *Brachycentrus* larvae (e.g., altered feeding behaviors, changed susceptibility to predation, reduced growth rates), such responses may result from longer-term exposure to various types of stressors [44].

Although landfill leachate contains a mix of substances that potentially may be toxic to aquatic life forms (i.e., heavy metals, organic compounds, ionized salts, ammonia), the toxicity of leachate from municipal solid waste landfills often has been attributed to un-ionized ammonia [4,8,9,10,11,12,13]. Leachate from the Winona County landfill contained a high concentration (247,000 µg/L) of ammonia nitrogen, along with high specific conductance, alkalinity, several salts, and chromium. These, either separately or in combination, were likely responsible for the toxicity observed in this study. We have no direct proof that ammonia was the primary toxic component of Winona County landfill leachate, as we did not perform any specific manipulations to isolate ammonia (e.g., pH adjustment to alter un-ionized ammonia ratios without removing other contaminants). Although past studies have concluded that ammonia may be the most likely toxic substance in landfill leachate, especially when alkalinity also is high [9,10], we are unable to make the same conclusion based on our study results.

We attempted to reduce ammonia concentrations in leachate in one test by pre-aerating the leachate prior to testing but observed no significant reductions in toxicity in 24 h experiments with *Brachycentrus* caddisfly larvae. Air stripping [2] or other biological treatments [2,4,5] typically are used to break down and detoxify landfill leachate. Ammonia stripping can be efficient and highly effective and is the most widely applied treatment of landfill leachates [2]. Our 24 h tests did not demonstrate statistically significant benefits of pre-aeration (to potentially remove toxic ammonia), although the similar LC50 values for all testing time periods (24 h up to 7 days) may suggest that more extended aeration of leachate (as was performed in all tests from 24 h and beyond) may have served to reduce ammonia concentrations and thereby reduce the toxicity of leachate during the longer-period tests. Future toxicity testing could expose *Brachycentrus* caddisfly larvae or some other sensitive species to landfill leachate that has been pre-aerated for varying periods of time (e.g., 24, 48, 72, 96 h) prior to use, along with concurrent testing of ammonia concentrations within the aerated leachate, to assess whether air stripping can be effective in reducing the toxicity of leachate to aquatic organisms.

## 5. Conclusions

Based on the effects of leachate toxicity tests on locally collected benthic invertebrates, it appears that Winona County landfill leachate has the potential to impact the benthic communities in Burns Valley Creek. Field collections indicate that sites nearest the landfill had higher Hilsenhoff biotic indices than the site further removed from the landfill, suggestive of benthic community exposure to some form of organic pollutant(s) nearest the landfill. In contrast, benthic communities nearest to the landfill exhibited higher taxa richness and biotic integrity than invertebrates at the more remote site, suggesting that invertebrates nearest to the landfill have not been exposed to leachate either at all or in quantities insufficient to alter aquatic communities, despite past and current potential of leachate to seep into groundwater aquifers from unlined landfill waste cells. Although several taxa present in the stream can suffer complete mortality when exposed to raw, undiluted leachate, sensitive taxa such as *Brachycentrus* caddisfly larvae typically were among the most abundant organisms within various sites in Burns Valley Creek, suggesting no current problems from leachate pollution.

Laboratory tests indicated that reported tolerances of aquatic organisms may not be indicative of their sensitivities, specifically to landfill leachate. As a complex mixture of many different potential pollutants, the toxic effects of leachate could result from many substances, such as high concentrations of un-ionized ammonia, various salts, and heavy metals such as chromium. LC50 concentrations of leachate did not change greatly among tests of differing duration when test solutions were constantly aerated, suggesting that aeration might be utilized to partially reduce the toxicity of Winona County landfill leachate within on-site holding tanks.

We suggest that future toxicity assessments of various substances that have the potential to enter streams and rivers should utilize locally collected aquatic invertebrates as test organisms, in addition to those standardized test taxa mandated by environmental regulatory agencies. Such an approach will provide better understanding of the true impact of leachate on local aquatic habitats, if it were to enter those systems from a nearby landfill.

## Figures and Tables

**Table 1:** Tolerance ratings [21,22,23,24,25,26,27] and relative abundances (%) of benthic invertebrates collected from MBVC, WBVC, and EBVC.

Taxa	Tolerance Rating	MBVC	WBVC	EBVC
NON-INSECTS				
Amphipods	4	9.16	5.04	4.36
Isopods	8	7.78	18.11	30.21
Acari	4		0.05	
*Physella*	8	0.09	8.78	
Nematomorpha	5	0.09		
*Amnicola*	8		0.05	
Planarian	4		0.05	
Oligochaeta	7		1.39	0.28
Sphaeriidae	8		0.05	
INSECTS				
Mayflies				
*Baetis*	4	20.70	4.71	20.11
Caddisflies				
*Brachycentrus*	1	57.60	24.63	11.21
*Glossosoma*	0	0.55	1.52	5.28
*Hesperophylax*	4		5.04	0.93
*Hydropsyche*	4	1.28	2.40	3.15
*Cheumatopsyche*	4		0.51	
*Hydroptila*	4			0.09
*Limnephilus*	4		0.05	
*Chimarra*	3			0.09
*Rhyacophila*	0			0.09
Beetles				
*Optioservus*	4	2.11	25.83	9.64
*Helichus*	4	0.18	0.51	0.74
*Gyrinus*	4		0.05	
True flies				
Chironomidae	6		0.05	0.19
*Antocha*	3	0.09	0.28	
*Tipula*	3	0.18		0.28
*Dicranota*	3		0.69	0.37
*Simulium*	6	0.18	0.18	12.97
*Hexatoma*	3		0.05	
Total organisms		1092	2164	1079

**Table 2:** Benthic invertebrate metrics and Kruskal–Wallis test results comparing MBVC, WBVC, and EBVC. Values are means with standard deviations in parentheses. Density expressed as number/m^2^.

Metric	MBVC	WBVC	EBVC	K-W *H*	DF	*p*
Density	1820 (435)	3607 (1525)	1798 (401)	3.29	2	0.193
Taxa richness	9.0 (0.0)	16.3 (1.2)	12.3 (2.5)	6.82	2	0.033
Hilsenhoff index	2.48 (0.56) excellent	4.15 (0.32) very good	4.88 (0.39) good	7.2	2	0.0273
B-IBI	17 (3) poor	48 (3) fair/good	27 (8) poor/fair	6.82	2	0.033

**Table 3:** Composition of Winona County landfill leachate collected during September.

Analyte	Unit	Result	Reporting Limit
Alkalinity, Total	mg/L	2490	10
Arsenic	µg/L	17.6	2.0
Biochemical oxygen demand	mg/L	241	1
Cadmium	µg/L	0.2	0.2
Chloride	µg/L	215,000	200
Chromium	µg/L	598,000	3000
Copper	µg/L	<2	2
EH, Field	units	−39.2	2
Iron	µg/L	21,400	8
Lead	µg/L	49.0	1.0
Magnesium	µg/L	170,000	30
Manganese	µg/L	1810	3
Mercury	µg/L	<0.06	0.06
Molybdenum	µg/L	<9	9
Nitrate + Nitrite	µg/L	<50	50
Nitrogen, Ammonia	µg/L	247,000	80
pH	units	7.86	1.00
Potassium	µg/L	161,000	100
Sodium	µg/L	460,000	50
Solids, Total Dissolved	mg/L	3390	1
Solids, Total Suspended	mg/L	28	1
Specific conductance	µmhos/cm	5234	1
Sulfate	mg/L	87	4
Turbidity	NTU	148	0.1
Zinc	µg/L	52	7
*Volatile organics*			
Acetone	µg/L	94.2	6.0
Ethyl ether	µg/L	1.7	0.7
Methyl ethyl ketone	µg/L	39.9	5.0
Tetrahydrofuran	µg/L	16.6	5.0

**Table 4:** Twenty-four h landfill leachate toxicity tests of field-collected aquatic invertebrates.

Taxa	Tolerance Value *	*n*	Mortality %
Amphipods—*Gammarus pseudolimnaeus*	4	10	100
Isopods—*Asellus intermedius*	8	12	100
Virile crayfish—*Faxonius virilis*	6	6	100
Water bug—*Belostoma*	5	10	0
Water scorpion—*Ranatra*	5	2	0
Cranefly larvae—*Tipula*	3	4	100
Caddisfly larvae			
*Brachycentrus*	1	10	100
*Ptilostomis*	4	11	0
*Pycnopsyche*	4	20	60
*Limnephilus*	4	6	33

* 0–2 = pollution sensitive, 3–8 = pollution intermediate, 9–10 = pollution tolerant.

**Table 5:** Twenty-four h toxicities of varying leachate concentrations on *Brachycentrus* caddisfly larvae.

Leachate	Percent Mortality	
Concentration %	Test 1	Test 2
100	100	100
90	100	100
80	100	100
70	100	100
60	20	80
50	20	0
40	0	0
30	0	0
20	0	0
10	0	0
0	0	0

**Table 6:** LC50 values for *Brachycentrus* caddisfly larvae exposed to Winona County landfill leachate for differing time periods.

Duration	Median LC50
24 h	46.5% leachate
48 h	50.5% leachate
96 h	50.5% leachate
7 days	54.8% leachate

## Data Availability

The original contributions presented in this study are included in the article. Further inquiries can be directed to the corresponding author.

## References

[1] Ritter W.F. (2024). The history of landfills and landfill gas management in the U.S. Acad. Environ. Sci. Sust..

[2] Luo H., Zeng Y., Cheng Y., He D., Pan X. (2020). Recent advances in municipal landfill leachate: A review focusing on its characteristics, treatment, and toxicity assessment. Sci. Total Environ..

[3] Anand N., Palani S.G. (2022). A comprehensive investigation of toxicity and pollution potential of municipal solid waste landfill leachate. Sci. Total Environ..

[4] Thomas D.J.L., Tyrrel S.F., Smith R., Farrow S. (2009). Bioassays for the evaluation of landfill leachate toxicity. J. Toxicol. Environ. Health B.

[5] Abdel-Shafy H.I., Ibrahim A.M., Al-Sulaiman A.M., Okasha R.A. (2024). Landfill leachate: Sources, nature, organic composition, and treatment: An environmental overview. Ain Shams Eng. J..

[6] Friedland A., Relyea R., Courard-Hauri D. (2012). Environmental Science: Foundations and Applications.

[7] Cunningham W.P., Cunningham M.A. (2010). Environmental Science: A Global Concern.

[8] Cameron R.D., Koch F.A. (1980). Toxicity of landfill leachate. J. Water Pollut. Control Fed..

[9] Clément B., Merlin G. (1995). The contribution of ammonia and alkalinity to landfill leachate toxicity to duckweed. Sci. Total Environ..

[10] Isidori M., Lavorgna M., Nardelli A., Parrella A. (2003). Toxicity identification evaluation of leachates from municipal solid waste landfills: A multispecies approach. Chemosphere.

[11] Svensson B.-M., Mathiasson L., Mårtensson L., Bergström S. (2005). Artemia salina as a test organism for assessment of acute toxicity of leachate water from landfills. Environ. Monit. Assess..

[12] Pivato A., Gaspari L. (2006). Acute toxicity test of leachates from traditional and sustainable landfills using luminescent bacteria. Waste Manag..

[13] Fauziah S.H., Izzati M.N., Agamutha P. (2013). Toxicity on Anabas testudineus: A case study of sanitary landfill leachate. Procedia Environ. Sci..

[14] Przydatek G. (2019). The analysis of the possibility of using biological tests for assessment of toxicity of leachate from an active municipal landfill. Environ. Toxicol. Pharmacol..

[15] Kalka J. (2012). Landfill leachate toxicity removal in combined treatment with municipal wastewater. Sci. World J..

[16] Matejczyk M., Plaza G.A., Nalęcz-Jawecki G., Ulfig K., Markowske-Szczupak A. (2011). Estimation of the environmental risk posed by landfills using chemical, microbiological and ecotoxicological testing of leachates. Chemosphere.

[17] Kalčíková G., Zupančič M., Levei E.A., Miclean M., Englande A.J., Gotvajn A.Ž. (2015). Application of multiple toxicity tests in monitoring of landfill leachate treatment efficiency. Environ. Monitor. Assess..

[18] Budi S., Suliasih B.A., Othman M.S., Heng L.Y., Surif S. (2016). Toxicity identification evaluation of landfill leachate using fish, prawn and seed plant. Waste Manag..

[19] Ghosh P., Thakur I.S., Kaushik A. (2017). Bioassays for toxicological risk assessment of landfill leachate: A review. Ecotox. Environ. Saf..

[20] Gupta A., Paulraj R. (2017). Leachate composition and toxicity assessment: An integrated approach correlating physicochemical parameters and toxicity of leachates from MSW landfill in Delhi. Environ. Technol..

[21] Hilsenhoff W.L. (1975). Aquatic Insects of Wisconsin.

[22] Eddy S., Hodson A.C., Underhill J.C., Schmid W.D., Gilbertson D.E. (1982). Taxonomic Keys to the Common Animals of the North Central States.

[23] Merritt R.W., Cummins K.W. (1984). An Introduction to the Aquatic Insects of North America.

[24] Pennak R.W. (1989). Fresh-Water Invertebrates of the United States.

[25] Thorp J.H., Covich A.P. (1991). Ecology and Classification of North American Freshwater Invertebrates.

[26] Hilsenhoff W.L. (1982). Using a Biotic Index to Evaluate Water Quality in Streams.

[27] Hilsenhoff W.L. (1987). An improved index of organic stream pollution. Grt. Lakes Entomol..

[28] Wittman E., Mundahl N.D. (2003). Development and validation of a benthic index of biotic integrity (B-IBI) for streams in southeastern Minnesota. Winona State Univ. Floruit.

[29] Magner J.A., Vondracek B., Brooks K.N. (2008). Grazed riparian management and stream channel response in southeastern Minnesota (USA) streams. Environ. Manag..

[30] Kumar V., Sheoran O.P., Rani S., Malik K. (2020). Development of a web-based tool for probit analysis to compute LC50/LD50/GR50 for its use in toxicology studies. J. Appl. Nat. Sci..

[31] Waters T.F. (1980). Streams and Rivers of Minnesota.

[32] Troelstrup N.H., Perry J.A. (1989). Water quality in southeastern Minnesota streams: Observations along a gradient of land use and geology. J. Minn. Acad. Sci..

[33] Lenat D.R. (1993). A biotic index for the southeastern United States: Derivation and list of tolerance values, with criteria for assigning water quality ratings. J. N. Am. Benthol. Soc..

[34] Hill B.H., Herlihy A.T., Kaufmann P.R., Stevenson R.J., McCormick F.H., Johnson C.B. (2000). Use of periphyton assemblage data as an index of biotic integrity. J. N. Am. Benthol. Soc..

[35] Wang L., Lyons J., Simon T.P. (2002). Fish and benthic macroinvertebrates as indicators of stream degradation in urbanizing watersheds. Biological Response Signatures: Indicator Patterns Using Aquatic Communities.

[36] Karr J.R., Chu E.W. (2013). Restoring Life in Running Waters: Better Biological Monitoring.

[37] Clements W.H., Kiffney P.M. (1994). Integrated laboratory and field approach for assessing impacts of heavy metals at the Arkansas River, Colorado. Environ. Toxicol. Chem..

[38] Courtney L.A., Clements W.H. (2002). Assessing the influence of water and substratum quality on benthic macroinvertebrate communities in a metal-polluted stream: An experimental approach. Freshw. Biol..

[39] Clements W.H. (2004). Small-scale experiments support causal relationships between metal contamination and macroinvertebrate community responses. Ecol. Appl..

[40] Clark J.L., Clements W.H. (2006). The use of in situ and stream microcosm experiments to assess population- and community-level responses to metals. Environ. Toxicol. Chem..

[41] Clements W.H., Vieira N.K.M., Church S.E. (2010). Quantifying restoration success and recovery in a metal-polluted stream: A 17-year assessment of physicochemical and biological responses. J. Appl. Ecol..

[42] Clements W.H., Kotalik C. (2010). Effects of major ions on natural benthic communities: An experimental assessment of the US Environmental Protection Agency aquatic life benchmark for conductivity. Freshw. Sci..

[43] Kotalik C., Cadmus P., Clements W. (2021). Before-after control-impact field surveys and novel experimental approaches provide valuable insights for characterizing stream recovery from acid mine drainage. Sci. Total Environ..

[44] Mundahl N.D., Mundahl E.D. (2024). Potential influence of suspended sediments on the population dynamics and behavior of filter-feeding Brachycentrus occidentalis (Trichoptera: Brachycentridae) larvae in a southeastern Minnesota, USA, trout stream. Water.

